# Predictive model, miRNA-TF network, related subgroup identification and drug prediction of ischemic stroke complicated with mental disorders based on genes related to gut microbiome

**DOI:** 10.3389/fneur.2023.1189746

**Published:** 2023-05-26

**Authors:** Jing Shen, Yu Feng, Minyan Lu, Jin He, Huifeng Yang

**Affiliations:** ^1^The Affiliated Jiangsu Shengze Hospital of Nanjing Medical University, Nanjing, China; ^2^The University of New South Wales, Sydney, NSW, Australia; ^3^The University of Melbourne, Parkville, VIC, Australia

**Keywords:** ischemic stroke, mental disorders, gut microbiome, machine learning, qRT-PCR, diagnostic model, drug prediction, transcription factors ischemic stroke

## Abstract

**Background:**

Patients with comorbid schizophrenia, depression, drug use, and multiple psychiatric diagnoses have a greater risk of carotid revascularization following stroke. The gut microbiome (GM) plays a crucial role in the attack of mental illness and IS, which may become an index for the diagnosis of IS. A genomic study of the genetic commonalities between SC and IS, as well as its mediated pathways and immune infiltration, will be conducted to determine how schizophrenia contributes to the high prevalence of IS. According to our study, this could be an indicator of ischemic stroke development.

**Methods:**

We selected two datasets of IS from the Gene Expression Omnibus (GEO), one for training and the other for the verification group. Five genes related to mental disorders and GM were extracted from Gene cards and other databases. Linear models for microarray data (Limma) analysis was utilized to identify differentially expressed genes (DEGs) and perform functional enrichment analysis. It was also used to conduct machine learning exercises such as random forest and regression to identify the best candidate for immune-related central genes. Protein–protein interaction (PPI) network and artificial neural network (ANN) were established for verification. The receiver operating characteristic (ROC) curve was drawn for the diagnosis of IS, and the diagnostic model was verified by qRT-PCR. Further immune cell infiltration analysis was performed to study the IS immune cell imbalance. We also performed consensus clustering (CC) to analyze the expression of candidate models under different subtypes. Finally, miRNA, transcription factors (TFs), and drugs related to candidate genes were collected through the Network analyst online platform.

**Results:**

Through comprehensive analysis, a diagnostic prediction model with good effect was obtained. Both the training group (AUC 0.82, CI 0.93–0.71) and the verification group (AUC 0.81, CI 0.90–0.72) had a good phenotype in the qRT-PCR test. And in verification group 2 we validated between the two groups with and without carotid-related ischemic cerebrovascular events (AUC 0.87, CI 1–0.64). Furthermore, we investigated cytokines in both GSEA and immune infiltration and verified cytokine-related responses by flow cytometry, particularly IL-6, which played an important role in IS occurrence and progression. Therefore, we speculate that mental illness may affect the development of IS in B cells and IL-6 in T cells. MiRNA (hsa-mir-129-2-3p, has-mir-335-5p, and has-mir-16-5p) and TFs (CREB1, FOXL1), which may be related to IS, were obtained.

**Conclusion:**

Through comprehensive analysis, a diagnostic prediction model with good effect was obtained. Both the training group (AUC 0.82, CI 0.93–0.71) and the verification group (AUC 0.81, CI 0.90–0.72) had a good phenotype in the qRT-PCR test. And in verification group 2 we validated between the two groups with and without carotid-related ischemic cerebrovascular events (AUC 0.87, CI 1–0.64). MiRNA (hsa-mir-129-2-3p, has-mir-335-5p, and has-mir-16-5p) and TFs (CREB1, FOXL1), which may be related to IS, were obtained.

## 1. Introduction

Stroke is one of the leading causes of death and disability globally, of which about 87 percent is ischemic stroke (IS) (1). Most IS patients have one or more comorbidities (2). IS patients with comorbidities experience more severe defects, increased disability and hospitalization rates, and higher mortality rates (3). Post-stroke cognitive impairment and dementia (PSCID) are the main sources of post-stroke morbidity and mortality worldwide (4). Current studies have shown that 25–30 percent of IS survivors develop vascular cognitive impairment (VCI) or vascular dementia (VaD) immediately or later (5). Post-stroke depression (PSD) is a general mental health problem affecting about 33 % of IS survivors. PSD adversely affects recovery and rehabilitation of cognitive and motor impairment after stroke, significantly increasing recurrence chances of neurovascular problems (6). Anxiety disorders affect about 1/4 of IS patients (7), which hinders IS rehabilitation and prevents patients from resuming daily activities (8), but clinical trials have not produced any clear evidence to guide the treatment of post-stroke anxiety disorders (9). There is a corresponding association between obsessive–compulsive disorder and IS. According to a national longitudinal study by Chen et al., patients with obsessive–compulsive disorder have a higher risk of developing IS during follow-up compared with non-obsessive–compulsive disorder controls (10), but the correlation between the two is not clear. Odds of carotid revascularization after stroke are lower in patients with psychiatric disorders, especially those with schizophrenia, depression, substance use disorders, and multiple psychiatric diagnoses (11). In patients with schizophrenia, the presence of atopic disease increases the risk of ischemic stroke. The increased the number of atopic comorbidities, the heightened the risk of ischemic stroke (11). We require more clinical data to clarify the causal relationship between SC, gut microbes, and IS. However, our findings will help predict IS early through clinical genetic testing, as well as to predict the high incidence of IS in specific populations, such as schizophrenia. Additionally, our research will contribute to a better understanding of the genetic, immunological, and metabolic mechanisms underlying IS’s high incidence and dangerous prognosis.

Human body’s gut microbiome (GM) is the largest microbiome that plays an important role in regulating the immune system (12). In the mouse model, GM is also associated with the occurrence and sequelae of IS (13, 14). IS usually causes intestinal dysfunction, GM imbalance, intestinal bleeding, and intestinal septicemia, thus affecting the poor prognosis (15). More and more evidence shows that there is a correlation between GM and mental disorders, such as anxiety disorder, depression (16), schizophrenia (17), and so on. However, there is a lack of research on the relationship between GM and IS complicated with mental disorders. Therefore, this study is mainly through the analysis of five kinds of mental disorders (schizophrenia, depression, anxiety disorder, obsessive–compulsive disorder, and dementia) and IS in GM.

## 2. Materials and methods

### 2.1. Datasets

**Figure 1:**
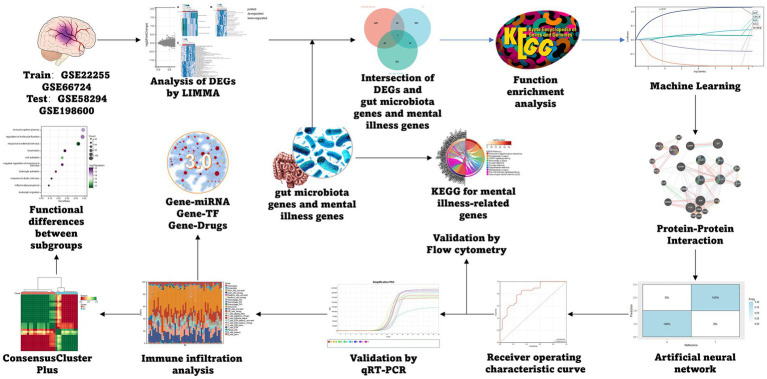
Flow chart.

The IS datasets GSE22255, and GSE66724 from the GEO database were selected as the training group (18).^1^ Merging multiple datasets required the use of the ‘inSilicoMerging’ algorithm from the R-software package (19). We utilized the Johnson et al. (20) method to eliminate the batch effect, to select GSE58294 and GSE198600 as the test group. Five genes related to mental disorders and GM were collected from Genecards, the NCBI database, and related literature. Finally, 710 genes related to mental disorders and 434 genes related to GM were obtained and sorted out according to different types (Supplementary Tables S1, S2). The process specific to this method is presented in Figure 1.

### 2.2. Differentially expressed gene screening

We used Limma (21), a generalized linear equation model to use as a difference table screening method. R-based Limma was utilized to analyze the differences in order to derive the DEGs among control and comparison groups. The criteria for identifying DEGs in this study were|log2 Fold Change (FC)| > 1 and *p* < 0.05, and the heat map and volcano plot of IS DEGs were visualized by sangerBox (22).

### 2.3. Gene Set function enrichment analysis

The DEGs of IS and related genes of mental disorders and GM were cross-screened by Venn plot. For functional enrichment analysis, the genes related to mental disorders of IS were obtained. Further, KEGG rest API^2^ and gene set function enrichment analysis (GSEA), were utilized to obtain the KEGG pathway’s latest gene annotation. Moreover, the GO annotation of the gene “org.Hs.eg.db:” in the R-package (vs. 3.1.0) (23) was used as the gene map background. The clusterProfiler from the R-package was utilized for enrichment analysis (24) to obtain gene enrichment results. For GSEA analysis (25), GSEA software (vs. 3.0) was used to divide the sample into two groups. Also “c2.cp.kegg.v7.4.symbols.gmt” subset from Molecular Signatures Database (26) was used to assess the molecular mechanisms and the related pathways. We preset the minimum gene set to 5 on the basis of gene expression profile and phenotypes groupings. The value of the maximum gene set was 5,000, and a *p*-value<0.05 and an FDR < 0.1was kept as indices of statistical significance.

### 2.4. Screening candidate genes related to is and mental disorders by machine learning and constructing a protein–protein interaction network

“Glmnet” (27) and “RandomForest” (28) in the R software package were used to integrate gene expression data with survival time and survival status. Further lasso-cox and Random Forest methods were utilized for regression analysis. Moreover, 10%-fold cross-validation was set up to derive the optimal model. The final diagnosis prediction model was obtained by cross-screening the outcomes of the two machine-learning techniques through the Venn plot. Protein–protein interaction (PPI) network was built using the Gene MANIA database. The latter is a user-friendly, flexible website for deriving assumptions about gene function, gene prioritization for functional analysis, and gene list analysis (29).

### 2.5. Validation of predictive models for diagnosis and prognosis

pROC (30) from the R package was used for ROC analysis to obtain AUC. Also, pROC’s CI function was utilized to assess the confidence interval (CI) and AUC so as to obtain the AUC result. Further, for visualization, sangerBox was used. Finally, we observed the expression of training set characteristic genes (GSE22255, GSE66724) and test group (GSE58294, GSE198600). In addition, a neuralnet (31) in the R software package was used to build an ANN for the characteristic genes obtained by the above method, thereby building a high-precision diagnostic model.

### 2.6. qRT-PCR and flow cytometry verification

Patients with acute IS hospitalized in Jiangsu Shengze Hospital, which is affiliated with the NMU (Nanjing Medical University) from January 1st, 2023, to January 15th, 2023, were enrolled retrospectively. Inclusion criteria: (1) the time of onset was within 7 days; (2) it met the diagnostic criteria revised by the Chinese Cerebrovascular Disease Classification 2015 of the Chinese Medical Association and was confirmed by head CT and/or MRI; (3) the medical records were complete. This research was conducted in accordance with the HD (Helsinki Declaration) and permitted by the Jiangsu Shengze Hospital’s Ethics Committee (Lun No.: 2022–017-01).

The qPCR gene of mRNA was detected in the PBMC samples of five patients with IS and five physical examiners. PBMC was extracted by the ficoll separation method (tbdscience, Tianjin, China), samples were anticoagulated by EDTA, and mRNA was extracted by magnetic beads method (BioPerfectus, Jiangsu, China). We used a one-step reverse transcription fluorescence quantitative PCR kit (BBI Lifesciences, Shanghai, China) for sybr green quantitative PCR amplification of mRNA. The primers were shown in Supplementary Table S3, and the amplification instrument was Applied Biosystems 7,500. The specificity of cDNA amplification was analyzed by melt curve, and the difference in gene expression was analyzed by Amplification Data.

We performed immunocytokine flow cytometry detection on the EDTA anticoagulated whole blood of 5 IS-confirmed patients and 5 physical examiners. An 8-item cytokine detection kit (multiplex microsphere flow immunofluorescence luminescence) (RAISEcare, Shandong, China) was used as the detection reagent, and BD FACSCanto II (Bccton, Dickinson and Company) was used as the cytokine detection instrument. The detection operation process is strictly in accordance with the kit instruction manual. We utilized flow cytometry to analyze the differences in the performance of the eight cytokines in the verification group.

### 2.7. Animal model and cell verification

Victoria G. Hernandez et al. induced stroke by distal middle cerebral artery occlusion (dMCAO) in an animal model and used RiboTag technology to obtain mRNA transcripts derived from astrocytes and microglia in the hyperacute phase (4 h) and acute phase (3 days) after stroke. The expression and log2 fold data for all sequenced genes are available on a user-friendly website (32).^3^

### 2.8. Immune infiltration analysis

The immune cell infiltration was analyzed by Cibersort (33) in R statistical package, and the correlation was evaluated by the spearman coefficient (34). The heat map of infiltrating immune cell correlation was drawn by corrplot (35) in the R software package.

### 2.9. Construction of miRNA and TF-hub gene network and drug prediction

The network of gene miRNA, gene-TFs, and gene-drug interaction was established by Network analyst (36).^4^

### 2.10. Subgroup analysis by candidate genes

Unsupervised hierarchical clustering analysis of IS samples was carried out utilizing the “ConsensusClusterPlus” of R (37) and the candidate genes’ expression as input information. For Gene Set Variation Analysis (GSVA), the R statistical package was utilized to assess each sample’s enrichment score in the gene set (38). The gene rank was predefined, and to evaluate the molecular mechanisms and related pathways, we downloaded the subsets c2.cp.kegg.v7.4.symbols.gmt, h.all.v7.4.symbols.gmt, and c2.cp.v7.4.symbols.gmt from Molecular Signatures Database. The minimum gene set was 5, and 5,000 was the maximum gene set. Each sample’s enrichment score in each gene set was evaluated, and finally, the enrichment score matrix was obtained. The DEGs of subgroups were obtained by Limma analysis, and the functional differences between subgroups were analyzed by KEGG and GO.

## 3. Results

### 3.1. Is differentially expressed genes’ screening

**Figure 2:**
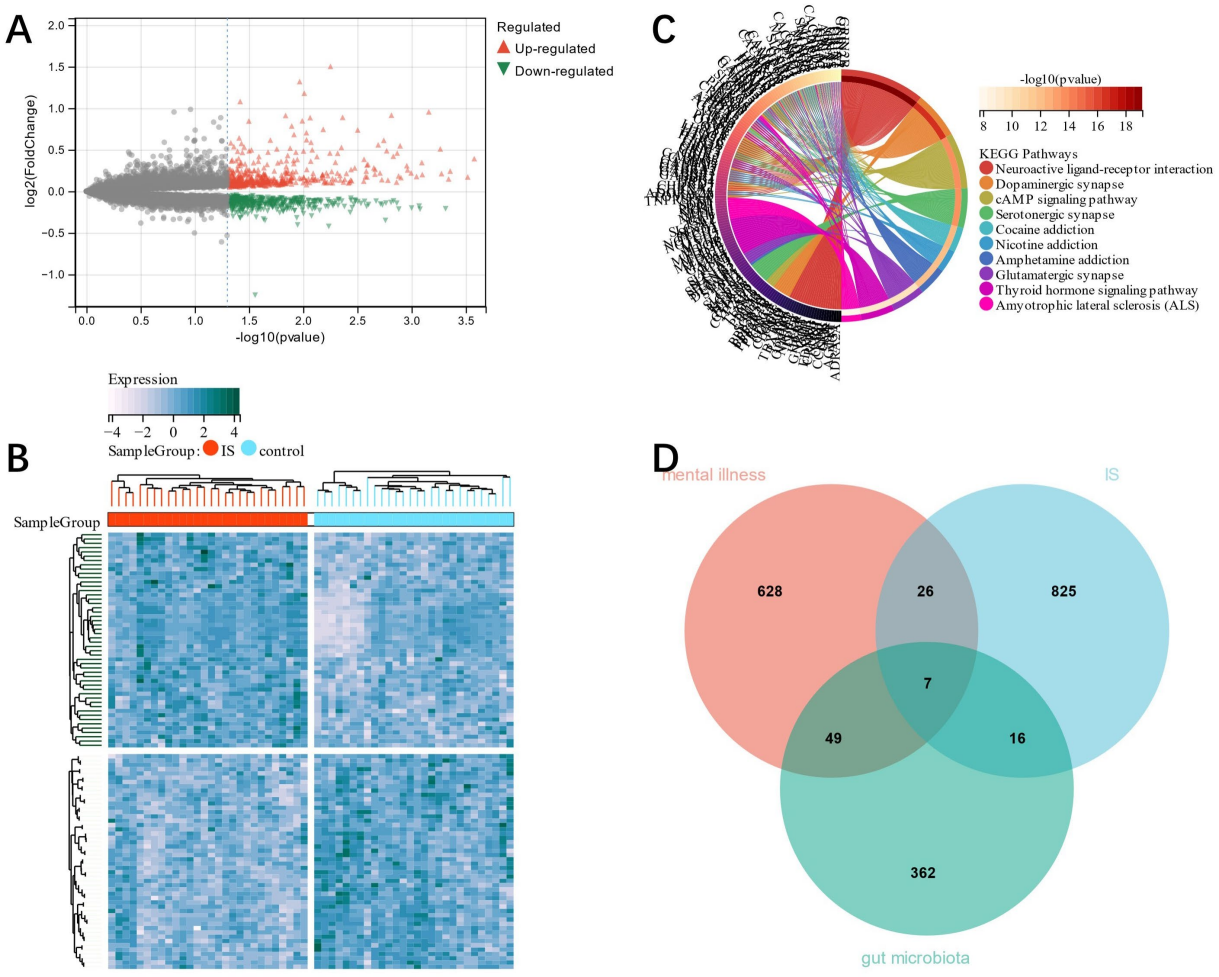
**(A)** volcano plot; **(B)** heat map of DEGs in IS; **(C)**, KEGG analysis corresponding to mental disorder-related genes; **(D)**, candidate genes were obtained by cross screening of mental disorder-related genes and GM in DEGs of IS.

Combining GSE22255 and GSE66724 as training group datasets, 874 DEGs were identified in IS training group dataset by the Limma method, from which 417 were down-regulated and 457 up-regulated (Figures 2A,B). The genes’ functional enrichment analysis that relates them to mental disorders was conducted. KEGG showed that genes related to mental disorders were mainly enriched in the interaction known as the Neuroactive ligand-receptor type (Figure 2C). This proved that there was a correlation between mental disorders and IS (39). Seven candidate genes related to mental disorders and GM were cross-screened by Venn plot (Figure 2D).

### 3.2. Functional enrichment analysis (FEA) of related candidate genes

**Figure 3:**
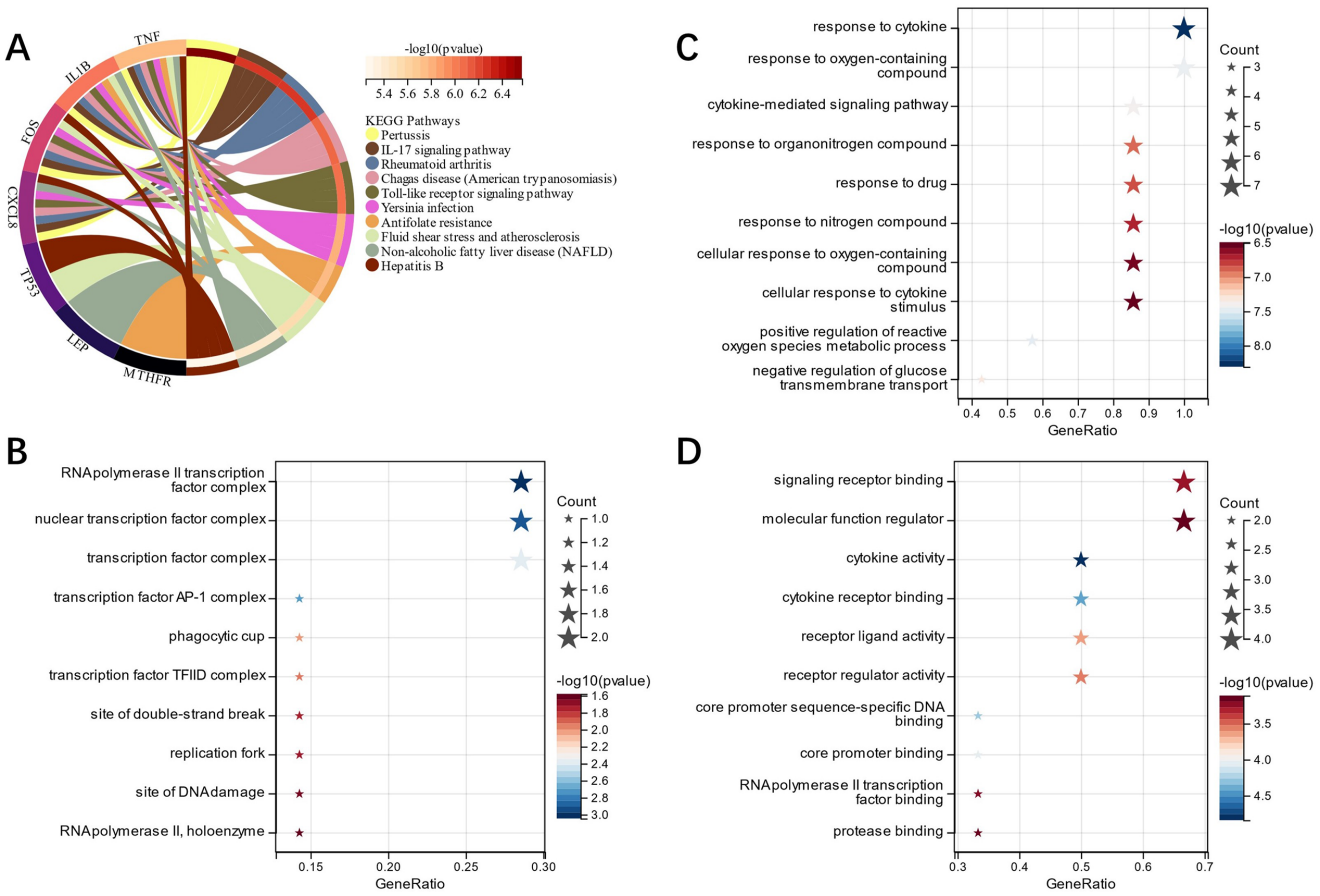
**(A)**: KEGG analysis of candidate genes; **(B)**: GO analysis of the cell component of candidate genes; **(C)**: GO analysis of biological process of candidate genes; **(D)**: GO analysis of the molecular function of candidate genes.

FEA of candidate genes was carried out, and KEGG analysis showed that the “Toll-like receptor signaling pathway,” “Rheumatoid arthritis,” “IL-17 signaling pathway,” and other pathways had enrichment of candidate genes (Figure 3A). GO analysis showed that in terms of cell composition (CC), the candidate genes were primarily located in the “RNA polymerase II transcription factor complex” and “nuclear transcription factor complex” (Figure 3B). The main biological processes (BP) of candidate genes included “response to cytokine,” “response to oxygen-containing compound,” and “cytokine-mediated signaling pathway” (Figure 3C). Molecular function (MF) analysis depicted that the most crucial processes among the candidate genes were “signaling receptor binding,” “cytokine receptor binding,” and “cytokine activity” (Figure 3D). Accordingly, our candidate genes may be involved in immune infiltration as well as pathways related to cytokines in IS.

### 3.3. Screening of candidate genes related to is and construction of PPI network and PCD By machine learning

**Figure 4:**
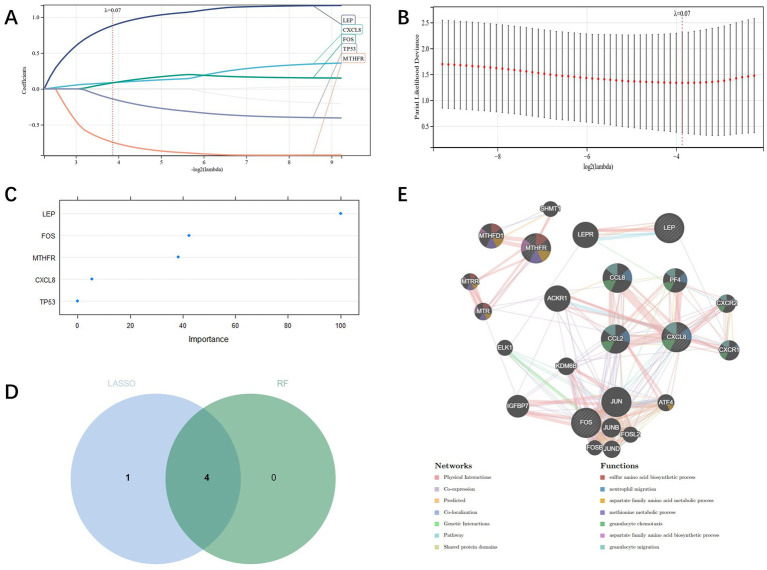
**(A,B)**: candidate genes’ screening through LASSO regression; **(C)**: Screening of candidate genes through RF regression; **(D)**: candidate genes cross screening through Venn plot and machine learning; **(E)**: PPI network construction of candidate genes.

Candidate genes were identified by LASSO regression, and the results depicted that five potential candidate genes were identified (Figures 4A,B). We also used RF regression to identify candidate genes and showed four potential biomarkers (Figure 4C). Then the results selected by the two kinds of machine learning were cross-analyzed, and ultimately four candidate genes (CXCL8, FOS, LEP, MTHFR) were obtained (Figure 4D). And the PPI network was established through these four candidate genes, of which Physical Interactions occupied 77.64%, Coexpression occupied 8.01%, and Predicted occupied 5.37% (Figure 4E).

### 3.4. Diagnostic model’s verification

**Figure 5:**
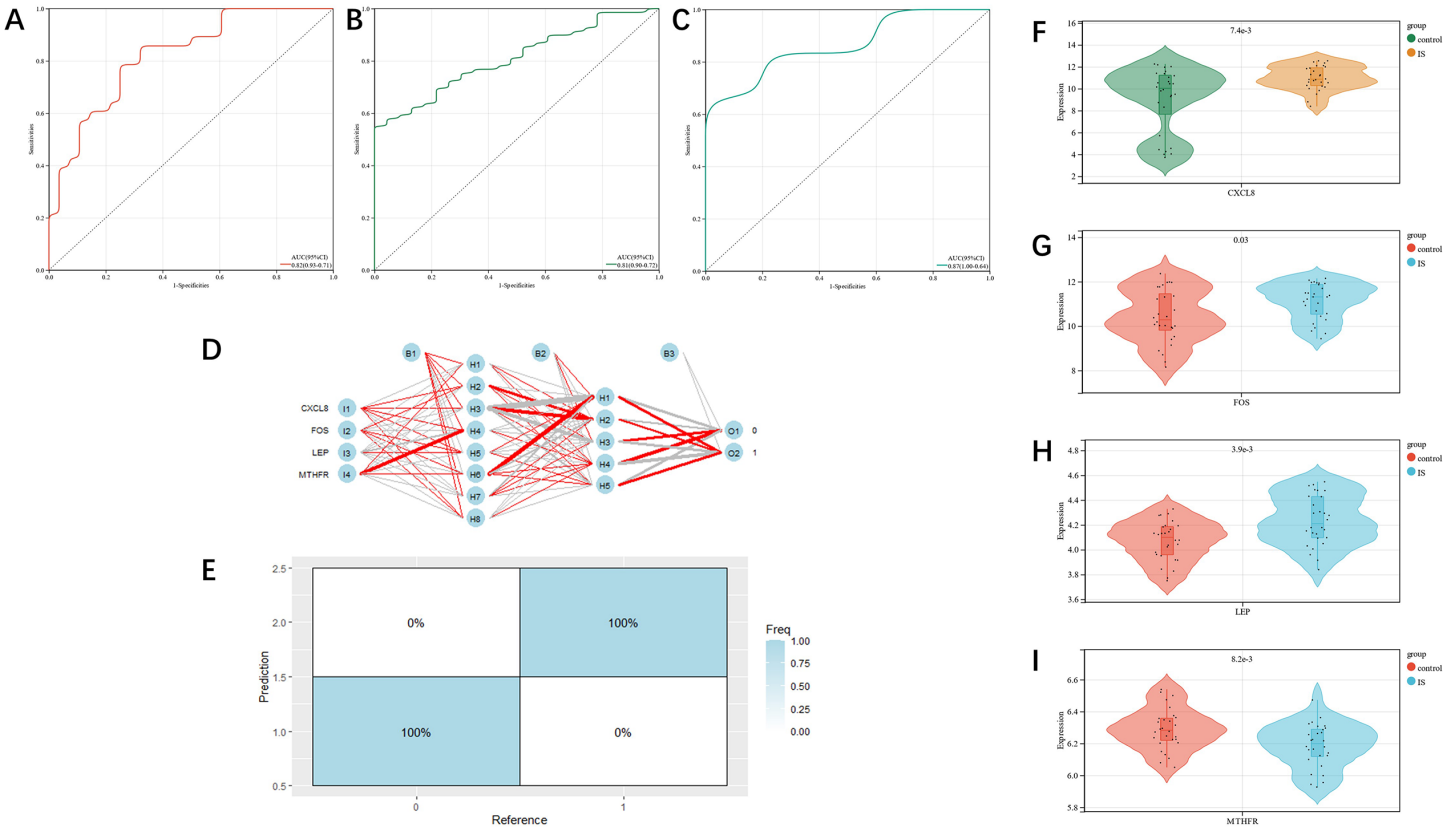
**(A)**: training group’s ROC curve; **(B,C)**: test group’s ROC curve; **(D,E)**: artificial neural network verification of training group; **(F–I)**: analysis of candidate gene expression profile in the training group (CXCL8, FOS, LEP, MTHFR, respectively).

**Figure 6:**
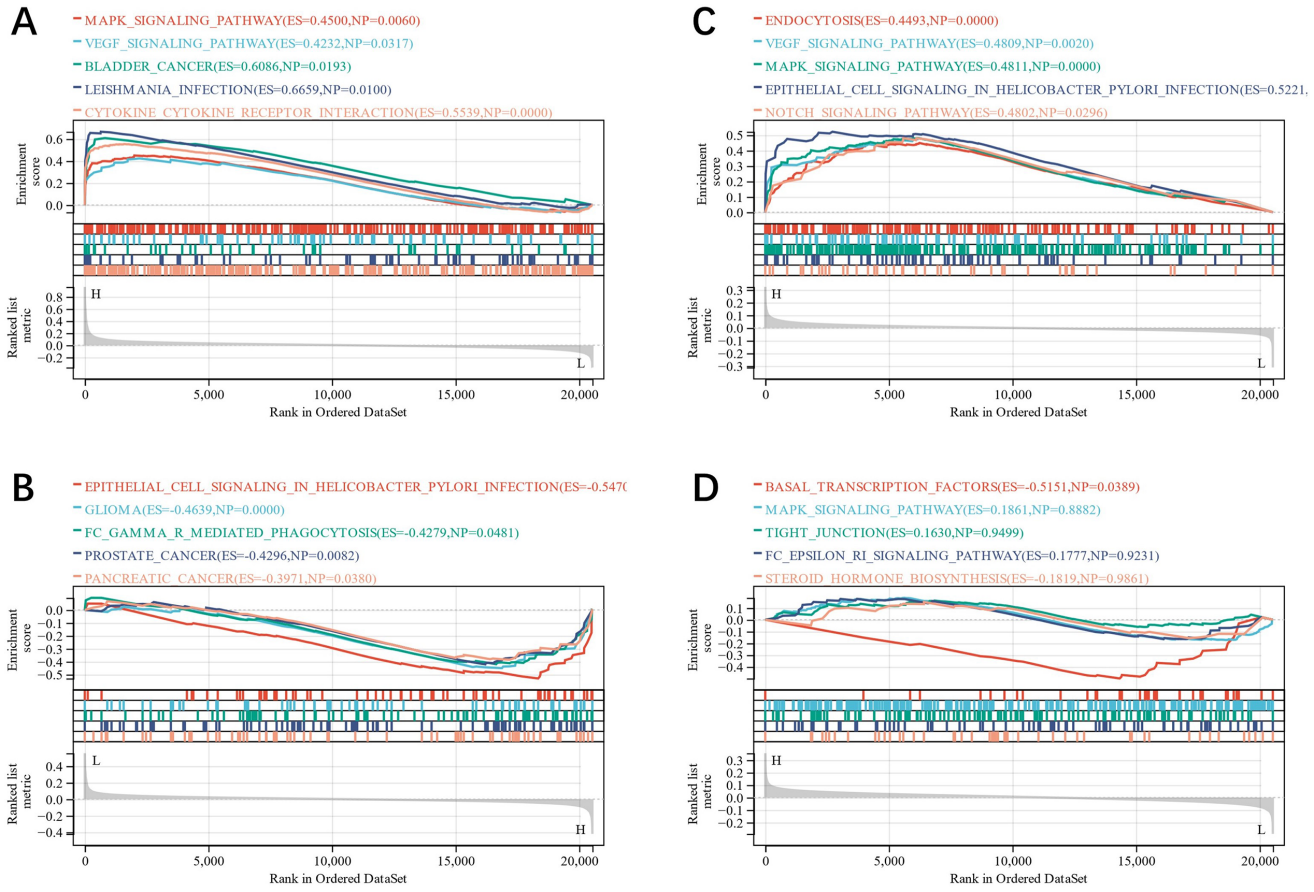
**(A–D)**: GSEA analysis of CXCL8, FOS, LEP and MTHFR.

The diagnostic value of the four candidate genes was verified by the ROC curve when all candidate genes were used as joint indicators (AUC 0.82, CI 0.93–0.71) (Figure 5A). We also put the diagnostic model into the verification group (GSE58294, GSE198600). It was shown that the diagnostic ROC (AUC 0.81, CI 0.90–0.72) of the positive and negative control groups in GSE58294 and the prognosis prediction ROC (AUC 0.87, CI 1–0.64) of the two groups in GSE198600 had a good predictive value (Figures 5B,C). The candidate genes were utilized to construct the neural network, and the outcomes depicted that the four candidate genes were able to distinguish the IS samples from the control samples, and the accuracy could reach 100% in the training group (Figures 5D,E). We also evaluated the expression profiles of the four candidate genes (Figures 5F–I), and the outcomes showed that there were statistically significant differences in candidate genes. GSEA analysis revealed that all four candidate genes were heavily enriched in immune-related pathways, such as the MAPK signaling pathway (Figures 6A–D). A relationship between candidate genes and pathways related to immune infiltration and cytokine production was further established by this study.

### 3.5. Qrt-PCR-based verification of candidate genes, cytokines validated by flow cytometry

**Figure 7:**
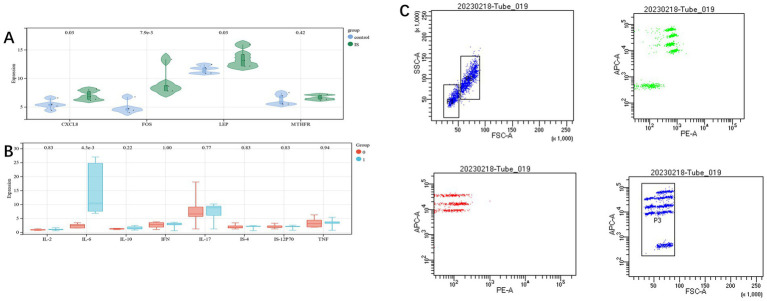
**(A)**: measuring the RNA expression of candidate genes in blood samples using qRT-PCR; **(B,C)**: differences of eight cytokines in the two groups.

In order to verify the reliability of the dataset, clinical samples were taken, and the expression level of candidate genes was further identified by qRT-PCR (see Supplementary Table S3 for specific data). CXCL8, FOS, and LEP revealed statistically significant differences (*p* < 0.05), but similar differences were not found in MTHFR, which may be due to the less number of samples. The overall results were similar to those of mRNA chips (Figure 7A).

Flow cytometry was used to detect cytokines in the two groups of cases.，we found a significant difference in IL-6 between the two groups (*p* < 0.05). This is consistent with the conclusion we predicted based on the GSEA analysis (Figures 7B,C).

### 3.6. Cell expression in animal models

**Figure 8:**
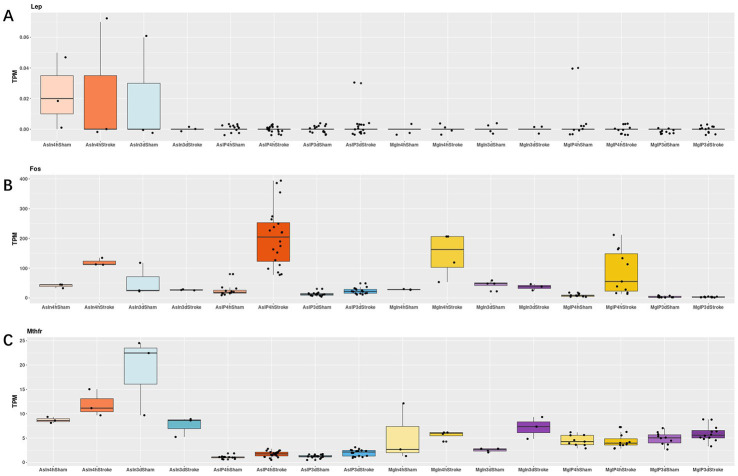
As = Astrocyte, Mg = icroglia, In = Input, IP=Immunoprecipitated, 4 h = 4 h, 3d = 3 days. **(A–C)**: the expressions of Lep, Fos and Mthfr in astrocyte cells and microglia cells of stroke group and sham group, respectively.

Regarding the Mthfr gene, we observed that in Astrocyte cells, the differences between the Stroke and Sham groups were 0.66 and 0.66 (Log2Foldchange) at 4 h and 3 days, respectively, with the Stroke group showing higher expression. In Microglia cells, there was no significant difference between the Stroke and Sham groups at 4 h, with a difference of −0.05, but at 3 days, the Stroke group showed higher expression with a difference of 0.61. For the Lep gene, there was no significant difference in expression at 4 h and 3 days in Astrocyte cells, with differences of −0.00 and − 0.02, respectively. For the Fos gene, in Astrocyte cells, there was a significant difference between the Stroke and Sham groups at 4 h, with a difference of 3.01, and at 3 days, with a difference of 0.88, both showing higher expression in the Stroke group. In Microglia cells, there was a significant difference between the Stroke and Sham groups at 4 h and 3 days, with differences of 3.35 and − 0.02, respectively, with the Stroke group showing higher expression (See Figure 8 and Table 1 for details).

**Table 1:** Lep, Fos and Mthfr in astrocyte cells and microglia cells of stroke group and sham group, respectively.

Gene	Log2Foldchange	FDR	Contrast	Sample	Timepoint	Cell
Mthfr	0.658273148	0.0015194	Stroke vs Sham	IP	4 hours	Astrocyte
Mthfr	0.666646781	9.739E-05	Stroke vs Sham	IP	3 days	Astrocyte
Mthfr	0.016733677	0.99996073	Stroke vs Sham	Input	4 hours	Astrocyte
Mthfr	−1.042966608	0.00022618	Stroke vs Sham	Input	3 days	Astrocyte
Mthfr	−0.052261005	0.92826721	Stroke vs Sham	IP	4 hours	Microglia
Mthfr	0.613049601	0.00128746	Stroke vs Sham	IP	3 days	Microglia
Mthfr	0.040883082	0.99999794	Stroke vs Sham	Input	4 hours	Microglia
Mthfr	0.532884857	0.16583881	Stroke vs Sham	Input	3 days	Microglia
Lep	−0.000231797	0.99996073	Stroke vs Sham	Input	4 hours	Astrocyte
Lep	−0.01781688		Stroke vs Sham	Input	3 days	Astrocyte
Fos	3.007177925	2.48E-34	Stroke vs Sham	IP	4 hours	Astrocyte
Fos	0.883801901	0.00049238	Stroke vs Sham	IP	3 days	Astrocyte
Fos	0.914864058	0.27297506	Stroke vs Sham	Input	4 hours	Astrocyte
Fos	−0.260105863	0.39797136	Stroke vs Sham	Input	3 days	Astrocyte
Fos	3.353981233	6.10E-13	Stroke vs Sham	IP	4 hours	Microglia
Fos	−0.021996332	0.97666485	Stroke vs Sham	IP	3 days	Microglia
Fos	1.902812386	0.00033217	Stroke vs Sham	Input	4 hours	Microglia
Fos	−0.247118725	0.43279267	Stroke vs Sham	Input	3 days	Microglia
Lep	−0.000231797	0.999960729	Stroke vs Sham	Input	4 hours	Astrocyte
Lep	−0.01781688		Stroke vs Sham	Input	3 days	Astrocyte
Fos	3.007177925	2.48E-34	Stroke vs Sham	IP	4 hours	Astrocyte
Fos	0.883801901	0.000492377	Stroke vs Sham	IP	3 days	Astrocyte
Fos	0.914864058	0.272975062	Stroke vs Sham	Input	4 hours	Astrocyte
Fos	-0.260105863	0.397971358	Stroke vs Sham	Input	3 days	Astrocyte
Fos	3.353981233	6.10E-13	Stroke vs Sham	IP	4 hours	Microglia
Fos	-0.021996332	0.976664854	Stroke vs Sham	IP	3 days	Microglia
Fos	1.902812386	0.000332169	Stroke vs Sham	Input	4 hours	Microglia
Fos	-0.247118725	0.432792674	Stroke vs Sham	Input	3 days	Microglia

As, Astrocyte, Mg = microglia, In = Input, IP=Immunoprecipitated, 4 h = 4 h, 3d = 3 days.

### 3.7. Immune cell infiltration analysis

**Figure 9:**
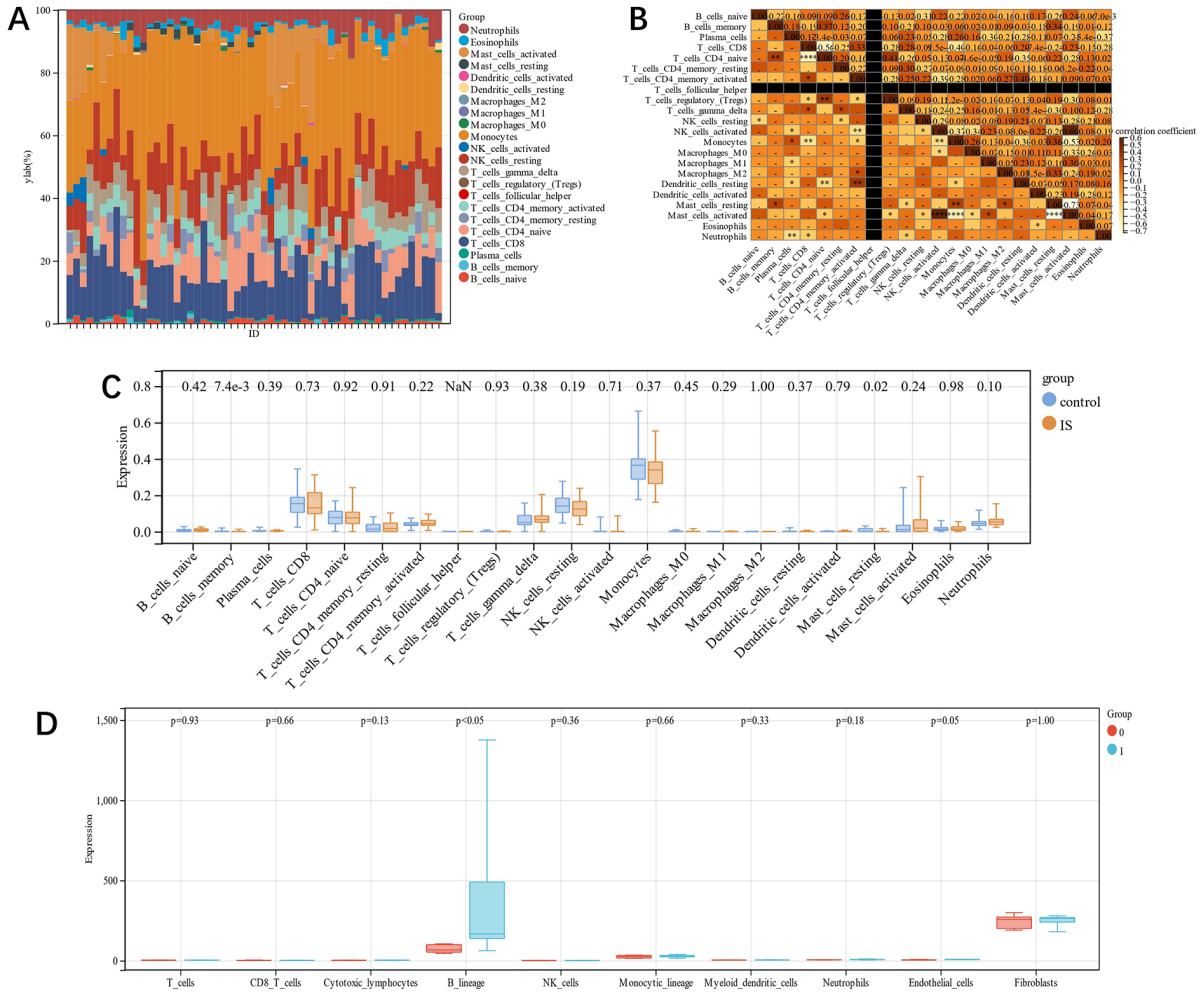
**(A)**: each sample’s relative percentage of 22 immune cells; **(B)**: The correlation among the 22 immune cells; **(C)**: immune infiltration difference between IS and control samples; **(D)**: Immune infiltration difference between the groups with and without carotid-related ischemic cerebrovascular events.

In this study, using the Cibersort algorithm, the concentration of 22 immune cells in IS samples and control samples in the training group was estimated (Figures 9A,B). The immune cell infiltration of IS and the control group was compared in the box plot (Figure 9C). The results revealed that there were statistically significant differences in memory B cells and resting mast cells in IS patients, and both were substantially compared to the control group. In the prognostic group of GSE198600, we found similar immune infiltration B lineage between the groups with and without carotid-related ischemic cerebrovascular events (*p* < 0.05) (Figure 8D).

### 3.8. Gene-miRNA, gene-TF, and gene-drug network diagram

**Figure 10:**
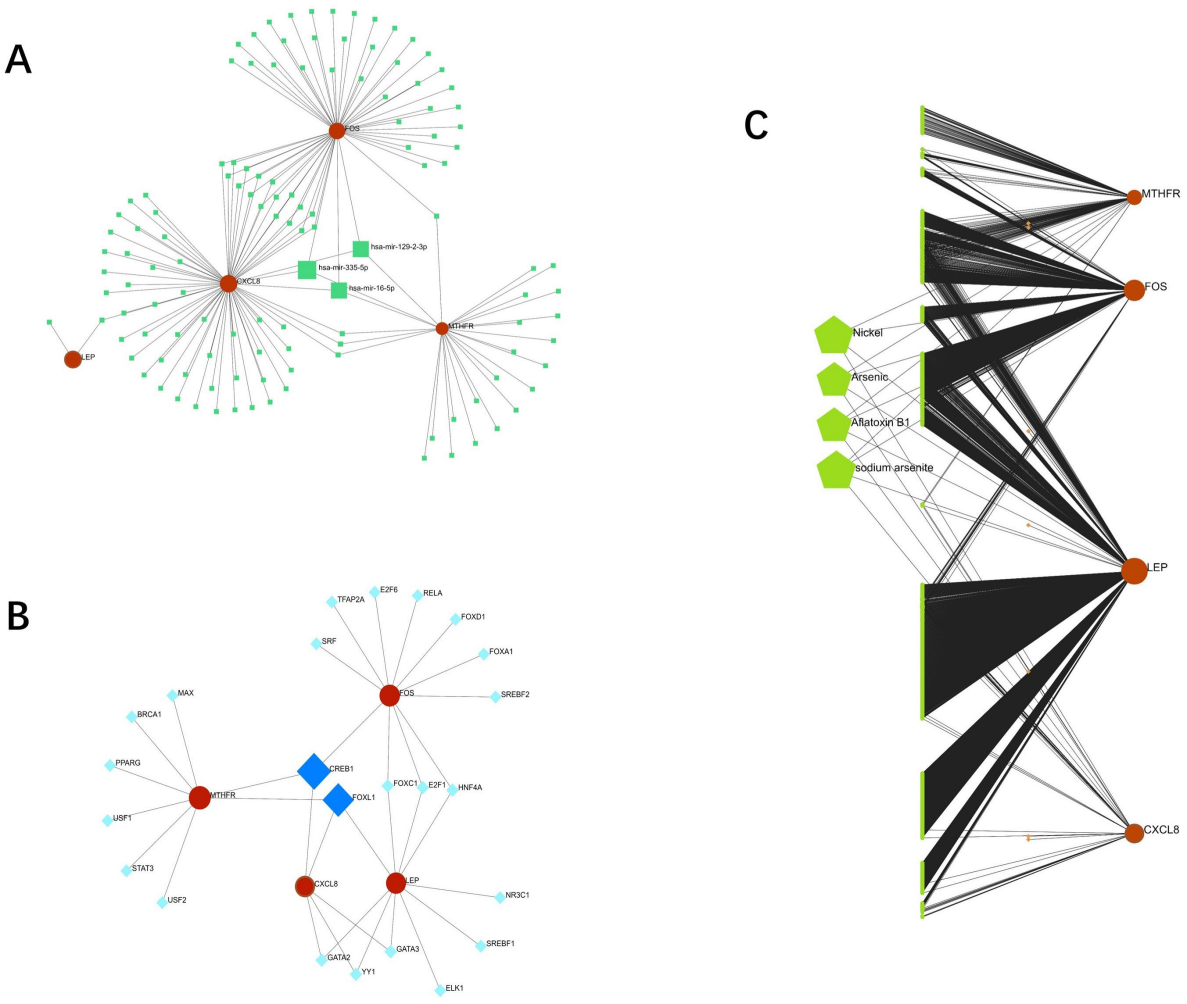
**(A)**: interaction between candidate genes and miRNA; **(B)**: Interaction between candidate genes and TFs; **(C)**: gene-drug interaction network (red represents candidate genes, orange data comes from DrugBank, green data comes from Comparative Toxicogenomics Database).

The interaction networks of genes and miRNA, genes and TF with genes and drugs were generated by Network analyst. Four candidate genes-miRNA networks were constructed, and it was found that hsa-mir-129-2-3p, has-mir-335-5p, and has-mir-16-5p could regulate the expression of CXCL8, FOS and MTHFR simultaneously (Figure 10A). Four candidate genes-TF networks were constructed, and the results revealed that CREB1 could regulate the expression of CXCL8, FOS, and MTHFR simultaneously, and FOXL1 could regulate the expression of CXCL8, LEP, and MTHFR simultaneously (Figure 10B).

Based on Drug Bank (40) and Comparative Toxicogenomics Database (41), a gene-drug interaction network was established (Figure 10C), and four of the most relevant drugs (Nickel, Arsenic, Aflatoxin B1, and sodium arsenite) were selected.

### 3.9. Candidate gene clusters’ consensus clustering (CC) analysis

**Figure 11:**
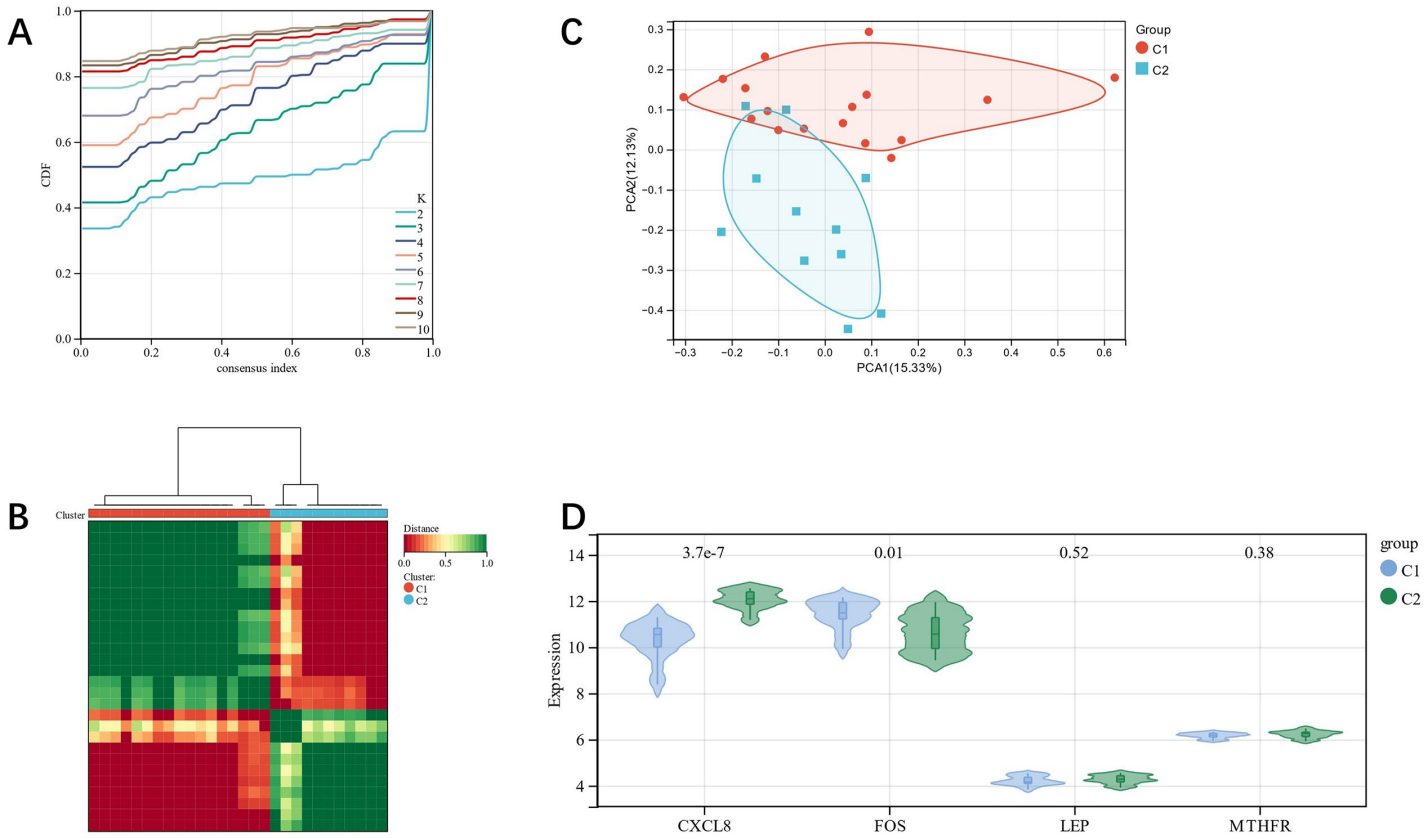
**(A,B)**: CC analysis of related candidate genes; **(C)**: PCA diagram shows subclusters’ distribution; **(D)**: violin diagram shows the differential expression of related candidate genes among subgroups.

By CC analysis of four related candidate gene models, we observed that there were the most substantial differences among different groups (Figures 11A,B), so they were divided into C1 and C2 categories. Using the PCA diagram, it was revealed that the gene expression patterns of different clusters were different (Figure 11C). The expression levels of related genes in the two subgroups were visualized by a violin diagram (Figure 11D). There was a statistically significant difference among the CXCL8 and FOS (*p* < 0.05).

### 3.10. GSVA of biological pathway among subsets of candidate genes

**Figure 12:**
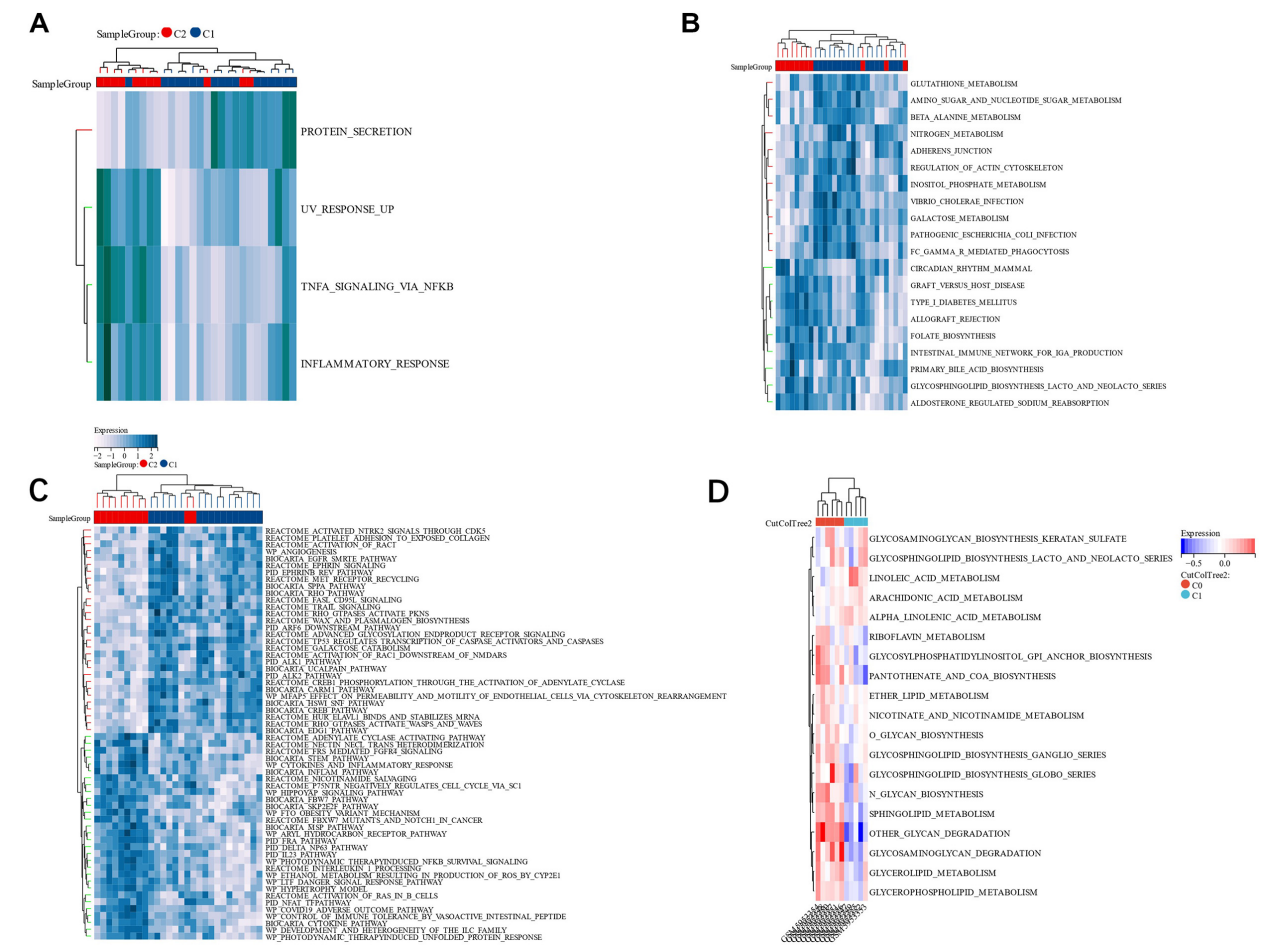
**(A)**: HALLMARK pathway’s GSVA; **(B)**: KEGG pathway’s GSVA; **(C)**: Reatcome pathway’s GSVA; **(D)**: A GSVA analysis of two groups in the validation set GSE198600 with different prognoses.

We found that TNFA signaling *via* NFKB, UV response up, and inflammatory response in group C1 was lesser compared to group C2, but protein secretion in group C1 was greater as compared to group C2 (Figure 12A). The KEGG pathways, including amino sugar, galactose metabolism, beta-alanine metabolism, and nucleotide sugar metabolism in group C1, were greater compared to group C2. However, the pathways of type I diabetes mellitus and Circadian rhythm in group C1 were lesser than those in group C2 (Figure 12B). In the Reatcome pathway, HuR (ELAVL1) binds and stabilizes mRNA, MET receptor recycling, and TP53 Regulates Transcription of Caspase Activators and Caspases in group C1 were significantly greater compared to group C2, but Cytokine Network and p75NTR negatively regulate cell cycle *via* SC1 were lower than those in group C2 (Figure 12C). The GSVA analysis of the two groups with different prognoses in the verification group GSE198600 revealed that they were very similar to the CC group, and they were significantly enriched in several pathways of the glycan metabolism (Figure 12D). Target genes are predictive of IS risk grouping in unknown situations, and the reasons for such grouping criteria may be related to psychiatric disorders, especially schizophrenia, and gut microbiota.

### 3.11. Functional differences among subgroups

**Figure 13:**
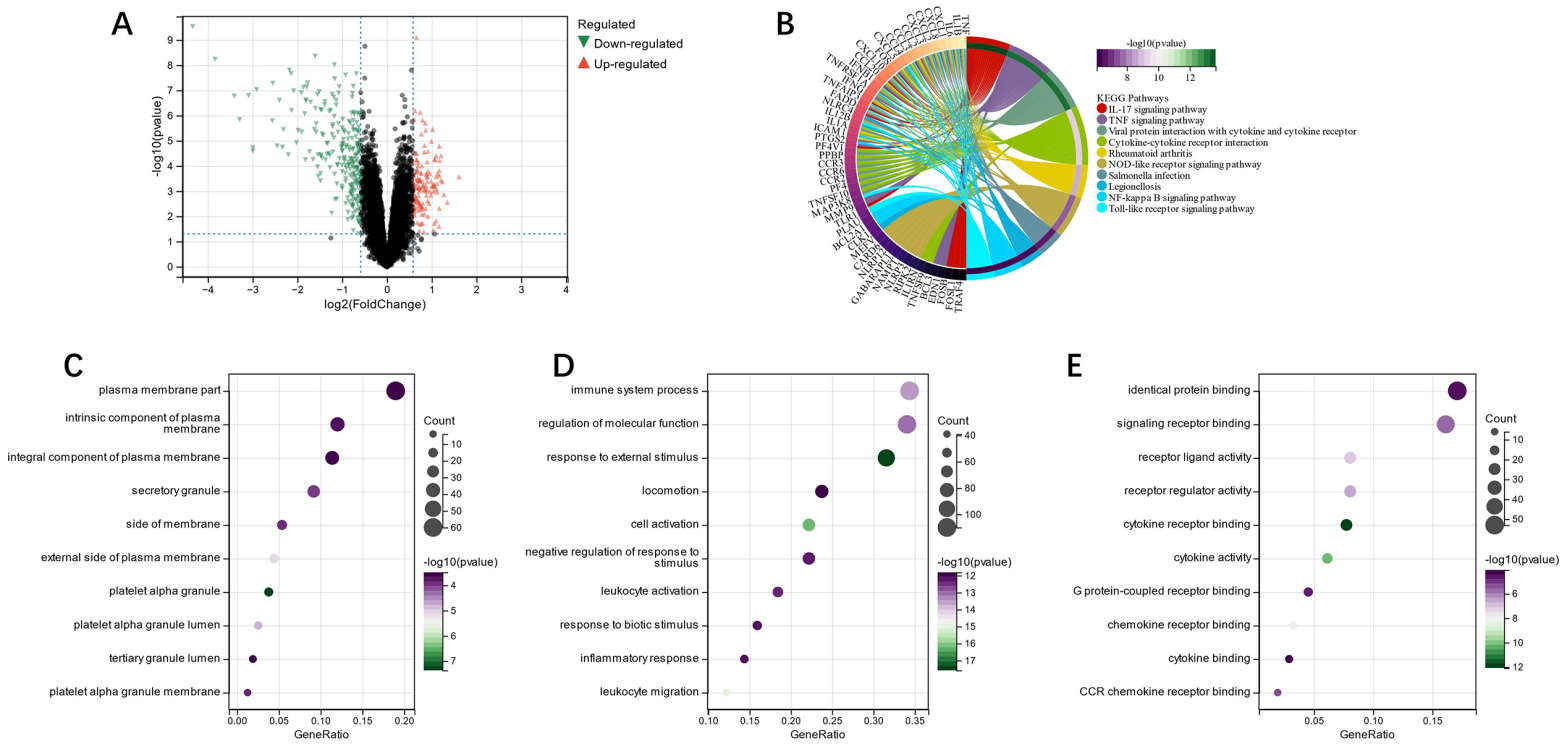
**(A)**: subgroup DEGs’ Volcano plot; **(B)**: differential genes’ KEGG analysis; **(C)**: differential genes’ GO analysis of cell composition; **(D)**: differential genes’ GO analysis of the biological process (BP); **(E)**: differential genes’ GO analysis of molecular function (MF).

Through Limma analysis, 375 DEGs were obtained, from which 237 were down-regulated and 138 were up-regulated (Figure 13A). FEA and KEGG analysis revealed that the enrichment of differential genes was primarily in the pathways “TNF signaling pathway,” “il-17 signaling pathway,” and “Cytokine-cytokine receptor interaction” (Figure 13B). GO analysis showed the differential genes were chiefly located in the “intrinsic component of plasma membrane” and “secretory granule” on the basis of CC (Figure 13C). The primary biological processes (BP) of differential genes include the “immune system process” and “regulation of molecular function” (Figure 13D). MF analysis revealed that the main processes of the differential genes were “identical protein binding” and “signaling receptor binding” (Figure 13E). Through GO enrichment and the KEGG analysis, we observed that these differential genes were primarily enriched in immune system-related pathways.

## 4. Discussion

Existing studies have shown that ischemic stroke (IS) with dementia, depression, and other mental illness symptoms are common. The gut microbiome (GM) plays a critical role in mental illness and IS (42). However, this study aimed to identify the differences between genes related to ischemic stroke (IS) and mental disorders in the gut microbiome (GM) through bioinformatics analysis and qRT-PCR verification, and to predict drugs related to IS through candidate genes. Four candidate genes (CXCL8, FOS, LEP, MTHFR), three miRNA (hsa-mir-129-2-3p, has-mir-335-5p, and has-mir-16-5p), and two TFs (CREB1, FOXL1) were identified, and the four most related drugs (Nickel, Arsenic, AflatoxinB1, and sodium arsenite) were obtained.

The results of this study suggest that the gut microbiome may play a critical role in mental illness and IS. CXCL8, FOS, LEP, and MTHFR were found to be potential candidate genes for IS. These genes have been previously linked to other diseases and pathways, including chemokine activity, interleukin-8 receptor binding, and metabolism of water-soluble vitamins and cofactors. In addition, several studies have shown a significant correlation between FOS and IS, and MTHFR gene polymorphism and the increased risk of IS. Our study supports these findings and provides further evidence of the role of these genes in IS.

Moreover, we found that IL-6, glucose metabolism, and B cell infiltration may be common pathways between schizophrenia and IS. This suggests that there may be a genetic correlation between these two diseases, and further studies are needed to clarify this relationship.

CXCL8 is a gene that codes for a protein and has been linked to diseases such as adult respiratory distress syndrome and melanoma. Pathways related to CXCL8 include TGF-pathway and MIF-mediated glucocorticoid regulation, as well as gene ontology annotations for chemokine activity and interleukin-8 receptor binding. Mouse experiments conducted by Hui Lv et al. suggest that CXCL8 may affect the development of IS by regulating the PI3K/Akt/NF-κB signaling pathway. Silencing CXCL8 led to a significant decrease in the deflection index, improved the size of the infarct, neurological function, and inhibited apoptosis index and glial cell loss (43). FOS is a gene that codes for a protein and is linked to diseases such as osteoblastoma and congenital systemic lipodystrophy. Pathways related to FOS include MyD88-dependent cascades initiated by endosomal and prolactin signal transduction. Gene ontology annotations for FOS include DNA binding to transcription factor activity and binding. Multiple bioinformatics analysis studies (44) have suggested a correlation between FOS and IS, and qRT-PCR verification has shown a statistically significant difference in IS-related FOS (*p* < 0.01).MTHFR is a gene that codes proteins. MTHFR is a gene that codes for proteins and is linked to diseases such as homocystinuria and folate-sensitive neural tube defects caused by a lack of N-methylenetetrahydrofolate reductase activity. Pathways related to MTHFR include the metabolism of water-soluble vitamins and cofactors, the methotrexate pathway (cancer cells), pharmacodynamics, and pharmacokinetics. Meta-analyses have shown a significant relationship between the C677T mutation of the MTHFR gene and the increased risk of IS. The MTHFR gene polymorphism is related to an increased IS risk, with a higher correlation observed in the Asian population (45). Ali Sazci et al. found that the MTHFR 1298C allele, C1298C genotype, and C677C/C1298C compound genotype are closely associated with ischemic stroke (46).

This study not only identified a genetic correlation between schizophrenia and IS, but also suggests that IL-6, glucose metabolism, and B cell infiltration are likely to be common pathways between these diseases. Four candidate genes were predicted, and the four most related drugs (Nickel, Arsenic, AflatoxinB1, and sodium arsenite) were obtained. Several studies have suggested a correlation between heavy metal levels and IS, with higher plasma concentrations of arsenic, aluminum, and cadmium and lower concentrations of iron and selenium increasing the risk of IS (47). Therefore, drugs containing Nickel, Arsenic, and sodium arsenite should be avoided in drug selection.

Further clinical data, particularly regarding schizophrenia and IS comorbidities and their follow-ups, are needed to clarify the causal relationship between SC, gut microbes, and IS. Relevant case collections and a large amount of clinical data and information will be needed to verify the conclusions of this study.

## 5. Conclusion

Through comprehensive analysis, a diagnostic prediction model with good effect was obtained. Both the training group (AUC 0.82, CI 0.93–0.71) and the verification group (AUC 0.81, CI 0.90–0.72) had a good phenotype in the qRT-PCR test. And in verification group 2 we validated between the two groups with and without carotid-related ischemic cerebrovascular events (AUC 0.87, CI 1–0.64). Furthermore, we investigated cytokines in both GSEA and immune infiltration and verified cytokine-related responses by flow cytometry, particularly IL-6, which played an important role in IS occurrence and progression. Therefore, we speculate that mental illness may affect the development of IS in B cells and IL-6 in T cells. MiRNA (hsa-mir-129-2-3p, has-mir-335-5p, and has-mir-16-5p) and TFs (CREB1, FOXL1), which may be related to IS, were obtained.

## Data availability statement

The original contributions presented in the study are included in the article/Supplementary material, further inquiries can be directed to the corresponding author.

## Ethics statement

The studies involving human participants were reviewed and approved by Jiangsu Shengze Hospital clinical laboratory. Written informed consent for participation was not required for this study in accordance with the national legislation and the institutional requirements.

## Author contributions

YF and JS wrote the article, ML, JH, and HY provided experimental help. All authors contributed to the article and approved the submitted version.

## Conflict of interest

The authors declare that the research was conducted in the absence of any commercial or financial relationships that could be construed as a potential conflict of interest.

## Publisher’s note

All claims expressed in this article are solely those of the authors and do not necessarily represent those of their affiliated organizations, or those of the publisher, the editors and the reviewers. Any product that may be evaluated in this article, or claim that may be made by its manufacturer, is not guaranteed or endorsed by the publisher.

## Footnotes

^1^www.ncbi.nlm.nih.gov/geo/

^2^https://www.kegg.jp/kegg/rest/keggapi.html

^3^https://buckwalterlab.shinyapps.io/AstrocyteMicrogliaRiboTag/

^4^https://www.networkanalyst.ca/
